# Terrestrial-aquatic connectivity structures microbial communities during the formation of thermokarst lakes

**DOI:** 10.1093/ismeco/ycaf027

**Published:** 2025-02-10

**Authors:** Martial Leroy, Melanie S Burnett, Isabelle Laurion, Peter M J Douglas, Cynthia M Kallenbach, Jérôme Comte

**Affiliations:** Centre Eau Terre Environnement, Institut national de la recherche scientifique, 490 rue de la couronne, Quebec City, QC G1K 9A9, Canada; Centre d'Études Nordiques (CEN), Université Laval, Pavillon Abitibi-Price, 2405 rue de la Terrasse, Quebec City, QC G1V 0A6, Canada; Centre d'Études Nordiques (CEN), Université Laval, Pavillon Abitibi-Price, 2405 rue de la Terrasse, Quebec City, QC G1V 0A6, Canada; Department of Earth and Planetary Sciences and Geotop, McGill University, 3450 University Street, Montreal, QC H3A 0E8, Canada; Centre Eau Terre Environnement, Institut national de la recherche scientifique, 490 rue de la couronne, Quebec City, QC G1K 9A9, Canada; Centre d'Études Nordiques (CEN), Université Laval, Pavillon Abitibi-Price, 2405 rue de la Terrasse, Quebec City, QC G1V 0A6, Canada; Centre d'Études Nordiques (CEN), Université Laval, Pavillon Abitibi-Price, 2405 rue de la Terrasse, Quebec City, QC G1V 0A6, Canada; Department of Earth and Planetary Sciences and Geotop, McGill University, 3450 University Street, Montreal, QC H3A 0E8, Canada; Natural Resource Sciences, McGill University, 21111 Lakeshore Road, Sainte-Anne-de-Bellevue, QC H9X 3V9, Canada; Centre Eau Terre Environnement, Institut national de la recherche scientifique, 490 rue de la couronne, Quebec City, QC G1K 9A9, Canada; Centre d'Études Nordiques (CEN), Université Laval, Pavillon Abitibi-Price, 2405 rue de la Terrasse, Quebec City, QC G1V 0A6, Canada

**Keywords:** microbial community structures, archaea, bacteria, assembly processes, thermokarst lakes, greenhouse gases, palsas, permafrost erosion, terrestrial-aquatic connectivity

## Abstract

Rising air temperatures and permafrost degradation drive the erosion of palsas (permafrost mounds mainly composed of frozen peat and ice layers) and lead to the formation of thermokarst ponds and lakes, known for their high greenhouse gas (GHG) emissions. This study investigates the impact of permafrost soil erosion during thermokarst lake formation on microbial community structure and its implications for GHG dynamics in a highly degraded permafrost valley (Nunavik, northern Quebec, Canada). Samples were collected from a palsa, an emerging lake connected to the palsa, surrounding peat and soil pore water, and two mature lakes which are older, stratified, and less connected to the palsa. Analysis of total and potentially active microbial communities, based on 16S rRNA gene amplicon sequence variants revealed significant changes in taxonomic and phylogenetic diversity during thermokarst lake formation. We found distinct assembly processes depending on the stage of formation. Firstly stochastics, they became more deterministic as lakes mature. Distinct methanogens/trophs communities in emerging lake led to lower CO_2_:CH_4_ ratio compared to the surface of mature lakes. Which presented a greater diversity of methanogens and distinct methanotrophic communities, with acetogenic, hydrogenotrophic and methylotrophic methanogens along anaerobic an aerobic methanotrophs. Multivariate analyses revealed that selection processes were primarily driven by concentrations of CH_4_, CO_2_, and NO_3_^−^. The interplay between the nitrogen and carbon cycles appears to be pivotal in these assemblages, with nitrogen playing key roles on community structure. These findings underscore the significance of terrestrial-aquatic connectivity in shaping microbial communities and GHG emissions in thermokarst lakes.

## Graphical Abstract

**Graphical Abstract:**
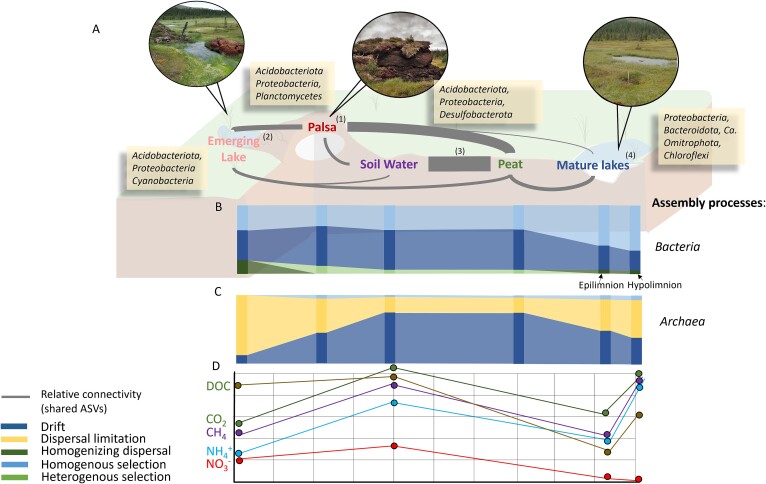

## Introduction

Subarctic and boreal peatlands are wetlands located in cold climates and permafrost-affected landscapes [1], making up nearly 90% of the world’s carbon-rich peatlands [2]. In these wet and cold conditions plant primary production exceeds decomposition, resulting in peat accumulation over centuries or millennia. In subarctic regions, permafrost exists in isolated patches, forming elevated mounds known as palsas, which rise a few meters above the peatland. With ongoing climate warming, palsas degrade into thermokarst lakes [3–6]. Thermokarst lakes and their surrounding wetlands play a crucial role in biogeochemical cycles including the production of greenhouse gases (GHG), such as carbon dioxide (CO_2_), methane (CH_4_), and potentially nitrous oxide (N_2_O) [5, 7–11]. Depending on the surrounding environment, organic matter (OM) transported to lakes may vary in age and availability, affecting microbial community structure and microbial GHG production [5, 12, 13].

Lakes closest to palsas are generally the most recently formed (i.e. emerging) [14] and are most directly affected by OM and nutrients (particularly nitrogen) inputs from permafrost soil and palsa erosion [15]. Mature lakes, typically located further away from palsas, receive much less palsa-derived OM. However, they may still receive old OM from deep peat deposits, as well as diverse contemporary OM sources such as mosses (Sphagnum) and other vegetation (e.g. *Carex aquatilis*) [13, 14, 16, 17].

There is hydrological continuity from palsas to mature lakes via wetland soils that are oversaturated with water [18]. We refer to these hydrological, physical, and biological exchanges as connectivity.

Previous studies conducted in subarctic thermokarst lakes have revealed an unsuspected high microbial diversity [14, 19–21] whose communities seem primarily driven by deterministic processes [20]. Yet, these studies have only investigated the lake microbial communities and therefore did not consider the influence of the surrounding landscape. The linkages between soil and lake microbial communities, particularly in these subarctic palsas and stratified lake systems have been overlooked. During the transition from soil to aquatic habitats, distinct microbial assembly processes shape microbial community structure. On the one hand, the high connectivity between terrestrial and aquatic habitats allows *mass effects*, where high microbial influx overrides environmental filtering, facilitating the dispersal of diverse taxa [22]. In the same way nitrogen enrichment have been linked to more stochasticity within a meta analyze comprising n-limited environment [23]. While on the other hand, selective pressure in aquatic environments strongly sort soil microbes, for instance, *homogeneous selection* fosters more uniform microbial communities through consistent environmental conditions [24, 25]. Stochastic processes, such as *dispersal limitation*, can also influence community structure, restricting microbial movement and allowing for random drift in population composition [26]. Previous studies have focused on microbial species sorting in Arctic and boreal lake ecosystems, but only a few studies have used a space-for-time approach to examine these dynamics across a gradient of permafrost degradation [12, 27–29].

In thermokarst landscapes, the link between community structure and GHG emissions is influenced by key microbial specialists such as methanogens and methanotrophs, which play essential roles in CH_4_ cycling [12]. However, the role of soil-aquatic connectivity in shaping these communities is still underexplored. Transitions from palsas to thermokarst lakes at various stages of formation represent a chronosequence of permafrost thaw, providing a unique framework for studying these ecological processes over time [6]. This framework enables us to investigate how microbial assembly processes in these transitions influence GHG dynamics and community structure, shedding light on the mechanisms behind microbial contributions to CH_4_ emissions.

Following the classification of pond and lake evolution based on Peura et al. (2020) [14]. We combined DNA and cDNA-based 16S rRNA gene sequencing with biogeochemical analyses to evaluate microbial community structures, assembly processes, and the role of connectivity across the different units in our study design. Our hypotheses are as follows: (i) Emerging, younger lakes directly connected to palsas exhibit a mass effect from soil microbes, while (ii) selection processes dominate in mature lakes, particularly shaping methanogenic communities, and (iii) stochastic processes are expected to prevail in other areas, such as palsa and peat.

## Materials and methods

### Study site and sampling

**Figure 1:**
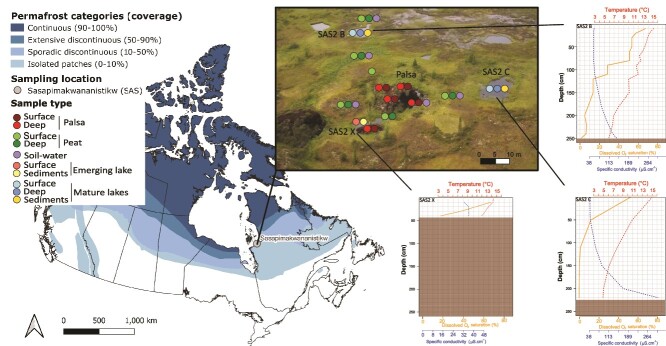
**Map of permafrost categories across Canada and the sampling site location in Nunavik, Canada.** Sampling locations are indicated by the dots and include the palsa, the adjacent wetland peat, the emerging lake (SAS2X), and the mature lakes (SAS2B and SAS2C), with their respective temperature, dissolved oxygen and specific conductivity profiles. The map was based on data from Natural Resources Canada, Atlas of Canada, 5th Edition [73].

The study area is located in a region of isolated permafrost in Nunavik, (northern Quebec), in a peatland draining into the Sasapimakwananistikw (SAS) river (Fig. 1). The peatland results from an accumulation of organic matter that began ~5800 years BP [30]. This accumulation was interrupted ~400 years BP with the Little Ice Age, when the establishment of permafrost caused the uplifting of palsas reaching 3 to 5 m in height. Thermokarst lakes began to form around 150 years ago with the degradation of permafrost and continue to expand today.

The sampling took place from August 13 to 17, 2021, during which three transects were investigated, two from a palsa to mature lakes (named SAS2 B and SAS2 C, respectively) 220 and 250 cm deep; and one from the same palsa to an emerging lake (SAS2 X, 48 cm deep) (Fig. 1). A total of 34 locations were sampled, including the palsa peat soils, surrounding wetland peat soils, lake water and lake sediments, with samples collected in three field replicates at each location. Soil and sediment sampling equipment were sterilized with 70% ethanol between samples, while the water sampling gear for DOC/DOM was burned at 550°C for 2 hours when using glass containers, all HDPE bottles were acid-washed overnight before being rinsed with MilliQ.

Palsa samples were collected using an 8-cm diameter corer (AMS Inc., Idaho, USA). Approximately 50 g each of homogenized soil from 5 cm and from 30 cm depth were collected separately, placed in sterile Whirl-pak bags (Nasco sampling LLC, Illinois, USA) and kept on ice until stored at −80°C at the CEN research station in Whapmagoostui-Kuujjuaraapik, within 24 h of collection. Peat samples were collected using a stainless-steel knife making 10 × 10 cm blocks, at the same two depths and treated the same way as above. Where possible, soil water from oversaturated peat was collected using a manual hand pump, stored in HDPE bottles in a cooler, and then filtered (see below).

Lake water samples were collected using a Van Dorn bottle, stored in HDPE bottles, and filtered within 24 h at the CEN research station. Water samples from the two mature lakes were collected from the surface and 20 cm above the bottom sediments (i.e. at 200 cm depth for SAS2B and 230 cm depth for SAS2C), and, when possible, from 50 cm and 150 cm depths. The emerging lake was less than half a meter deep, so it was sampled only at the surface and 20 cm above the bottom. For soil pore-water and lake water samples, 300 ml (or up to clogging) were filtered onto Sterivex filtration units (0.2 μm pore size, MiliporeSigma, Germany) using sterile syringes, and then immediately stored at −80°C in Whirl-Pak bags. Lake sediments were also sampled using a van Veen sediment grab sampler and were stored and processed the same way as soil samples.

### Environmental data

Dissolved oxygen (DO) concentration (% saturation relative to the atmosphere), temperature (°C), and specific conductivity (Cond, in μS.cm^−1^) were measured at each sampling site at the same time as sample collection, with a proODO probe (YSI Inc., Ohio, USA). At the field station laboratory, lakes and soil water samples were prepared for subsequent measurements of inorganic nutrients, including concentrations of NO_3_^−^ + NO_2_^−^ (abbreviated NO_3_^−^ hereafter), NH_4_^+^ (μgN.L^−1^), and PO_4_^3−^ (μg P.L^−1^); filtrates from the Sterivex were used for this purpose. A volume of 60 ml collected in HDPE bottles were kept frozen until they were analyzed at the laboratory with a Lachat Quikchem Flow Injection Analysis System (HACH, Colorado, USA) for NO_3_^−^ and NH_4_^+^, while PO_4_^3−^ was measured with an Astoria 2 Analyser (Astoria-pacific, Oregon, USA). Total phosphorus (TP) and total nitrogen (TN) were analyzed on unfiltered samples fixed at 0.1% final concentration of H_2_SO_4_, after a digestion step and measured with the Astoria 2 Analyser. Dissolved organic carbon (DOC; μg C.L^−1^) was quantified on filtered samples with a Sivers M9 TOC analyzer (Water Technologies & Solutions, Pennsylvania, USA). GHG concentrations (CO_2_, CH_4_, N_2_O) in lake and soil water were measured from samples collected *in situ* in 12 ml Exetainers (Labco, UK) using the headspace method for gas extraction. Additional details and calculations are provided in the SI. The CO_2_:CH_4_ ratio of the concentrations was calculated as it may be more responsive to differences in methanogenesis or methanotrophy.

### Microbial community analyses via metabarcoding and flow cytometry

To characterize the bacterial and archaeal communities, soil, sediments, and water DNA and RNA were coextracted in two distinct fractions using the Zymobiomics DNA/RNA microprep extraction kit (Zymo Research Corp., California, USA), following the manufacturer’s instructions. Extraction controls were done on ultrapure water (MilliQ) filtered on Sterivex units and processed in the same manner as the samples.

Extracted RNA was preserved at −80°C pending cDNA synthesis. Retro Transcriptase cDNA synthesis was performed on ice, with the High-Capacity cDNA Reverse Transcription Kit (AppliedBiosystems, California, USA), template concentration was adjusted to reach a minimum of 20 ng of RNA, then performed according to manufacturer protocol. The 16S rRNA gene amplicon sequencing of the V4-V5 region, for both DNA and cDNA templates was performed on an Illumina MiSeq platform (Illumina, Inc. California, USA) at Integrated Microbiome Resource IMR (University of Dalhousie) using the universal primers (515FB = 5’-GTGYCAGCMGCCGCGGGTAA-3′ and 926R = 5’-CCGYCAATTYMTTTRAGTTT-3′) known to cover a wide range of bacteria and archaea [31]. Primers were removed using cutadapt (v. 3.5) [32], where reads from the DNA and cDNA templates were trimmed to 275 bp for forward reads and 225 bp for reverse reads, plus an additional trimmed 5 bp on each side, and then filtered for quality at a max EE = 3. Sequence reads were assigned as amplicon sequence variants (ASVs) using the DADA2 [33] pipeline against the Silva database (v138.1) [34] in R software (version 4.2.2) [35]. After all quality check, trimming and filtering steps, including collapsing 100% identical ASVs (*collapseNoMismatch,* DADA2) and removing of *Chloroplasta* like sequences, a total of 1 502 735 sequences remained (20 561 ASVs) for a total of 98 samples that were further analyzed in this study. For the cDNA template, a total of 976 061 sequences in 58 samples (21 856 ASVs).

For both DNA and cDNA, unique sequences were aligned with Muscle5 (v. 5.1), a multiple sequence alignment software [36] using the super5 algorithm [36]. The aligned sequences were processed in FastTree (v. 2.1.11) [37] to construct a maximum likelihood phylogenetic tree using nearest-neighbor interchanges and subtree-prune-regraft moves [38]. The retained nucleotide evolution model was GTR + CAT. A gamma distribution of evolutionary rates [39] was used to rescale branch lengths. The phylogenetic tree were further used to calculate weighted UniFrac distance matrix [40] implemented in the *phyloseq* package [41]. The weighted UniFrac distances for DNA and cDNA templates were performed on the unrarefied dataset as it is weighted by the relative abundance and is robust to differences in library sizes [42]. The weighted UniFrac distance matrices were further plotted as Multidimensional scaling plot (MDS, i.e. Principal Coordinates Analysis -PCoA-) with uncertainties ellipses at 0.95. PCoA and composition plots visualization was perform using *microViz* and *microeco* packages [43, 44]. Surface was added on the MDS with *ordisurf* from vegan package [45], as the ratio of observed dissolved CO_2_ concentration and dissolved CH_4_ concentration is soil water and lakes water samples.

In addition, microbial flow cytometry was used to assess population shifts in optical characteristics such as side scatter (SSC) and forward scatter (FSC), which serve as proxies of cell complexity and size. The methods and gating strategies are provided in the SI.

### Calculation methods and statistical analyses

Samples were grouped according on their position in the transect, depth, and lake type (see Table 1 for detail). Two PERMANOVA analyses (vegan package) were conducted on weighted UniFrac distance matrices following Buttigieg and Ramette (2014) [46]. The first PERMANOVA included all samples, using classification as the explanatory variable (R^2^ = 0.56, *P* < .01). The second focused on lake and soil water samples, testing all combinations of environmental variables, with AIC values calculated using the *AICcPermanova* package [47]. The best explanatory model, with the lowest AICc, included DO, NH₄^+^, NO₃^−^, PO₄^3−^, TN, CH₄, CO₂, DOC, CN ratio, Template. A dbRDA was performed on Hellinger-transformed abundances to examine relationships with physicochemical data, including GHG concentrations. Collinearity tests led to the removal of total TP and temperature (T) due to high correlation with TN and DO, respectively. Missing values were handled using k-nearest neighbors (k = 8, *DMwR*2 package [48]). A second PERMANOVA was conducted on this matrix.

**Table 1:** Metabarcoding sample number per category of samplings.

**Type**		***n* sample DNA**	***n* sample cDNA**
Palsa	Surface	15	4
	Deep	12	9
Peat	Surface	16	9
	Deep	12	9
Soil water	-	11	7
Emerging lake	Water	6	5
	Sediments	3	2
Mature lake	Surface	11	7
	Deep	9	5
	Sediments	3	1

Alpha diversity metrics (observed richness, Chao1, Shannon Index) were calculated on rarefied dataset (5000 ASVs) using the *phyloseq* package. Significance was assessed using the Kruskall-Wallis test for overall differences, followed by pairwise Wilcoxon tests between each group. P-values were corrected for false discovery rate using the Benjamini-Hochberg adjustment method.

Microbial community assembly processes were analyzed using iCAMP [49], which applies a null model for each phylogenetic bin. Deterministic processes were identified when the absolute value of the Beta Net Relatedness Index (βNRI) exceeded 1.96, with homogeneous selection indicated by βNRI < −1.96 and heterogeneous selection by βNRI >1.96. The Raup-Crick (RC) metric was used to classify stochastic processes: RC < −0.95 indicated homogenizing dispersal, RC > 0.95 indicated dispersal limitation, and |RC| > 0.95 suggested drift. For the bacterial community analysis, *icamp.big* function was run with a *bin.size* of 24, using the Hellinger transformation method, the default phylogenetic metric (*bMPD* or *bNRI*), and the *sig.index* parameter as *SES.RC* (meaning a significance index based on standardized effect size for phylogenetic measures) and the modified Raup-Crick (*RCbray*) was used for taxonomic metrics. For the archaeal community, the same approach was applied, but with a bin size of 12. In both cases, the resulting iCAMP metric was *bNRIiRCbraya*. Bootstrapping (1000 iterations) was used to estimate the variation in the relative importance of each process within each group, followed by a comparison of differences between groups, as implemented in the *icamp.boot* function.

Finally, the relative proportions of each assembly processes between samples were transformed into a distance matrix using the *col3.dist* function in iCAMP. A Mantel test (Spearman correlation, 999 permutations) was then performed for each environmental parameter using distance matrices based on Euclidean distance.

## Results

### Environmental characteristics along the sampled transects

The emerging lake (SAS2 X) was in close contact with the remains of the collapsed palsa. This shallow lake was partially stratified at time of sampling; although a sharp decrease in oxygen with depth was observed (and a small thermal structure), anoxic conditions were not reached at the bottom (Fig. 1). This lake also showed higher NO_3_^−^ concentrations (on average 12.9 μg N.L^−1^) as compared with the mature lakes (6.5 and 2.9 μg N.L^−1^ for surface and hypolimnetic averages respectively; Table 2). The mature lakes are much deeper and thus showed stronger stratification of the water column in terms of temperature, oxygen, and conductivity (Fig. 1). Mature lakes also presented pronounced NH_4_^+^ gradients, with surface concentrations 15 to 50 times lower than those at the bottom (average of 1038.8 μg N.L^−1^) whereas NO_3_^−^ levels were 2 to 4 times higher at the surface (Table 2). Soil water presented the highest concentrations of TN, TP, PO_4_^3−^, and NO_3_^−^ but lower NH_4_^+^ (on average 159.2 μg N.L^−1^) than hypolimnion of mature lakes (Table 2).

**Table 2:** Nutrient and greenhouse gas (GHG) concentrations along the transect from soil water to emergent and mature lakes. Mean and standard deviations are provided for ammonium (NH_4_^+^), the sum of nitrate and nitrite (NO_3_^−^), total nitrogen (TN), phosphate (PO_4_^3−^), total phosphorus (TP), dissolved organic carbon (DOC), and dissolved CH_4_, CO_2_ and N_2_O. The ratio CO_2:_CH_4_ is also included in the table.

**Solutes and GHG**	**Soil water**	**Emerging lake**	**Surface of mature lakes**	**Hypolimnion of mature lakes**
NH_4_^+^ (μgN.L^−1^)	159.2 ± 65.5	30.2 ± 17.3	66.1 ± 48.2	1038.8 ± 657.8
NO_3_^−^ (μgN.L^−1^)	15.6 ± 2.2	12.9 ± 1	6.5 ± 2.9	2.9 ± 1.9
TN (μgN.L^−1^)	29064.1 ± 23748.9	1524.8 ± 399.8	614.8 ± 182.5	1943.9 ± 915.5
PO_4_^3−^ (μgP.L^−1^)	20.6 ± 8.9	3.4 ± 1	3.5 ± 0.8	2.0 ± 0.2
TP (μgP.L^−1^)	1512.9 ± 122	54.8 ± 23.4	15.2 ± 10.6	40.8 ± 24.9
DOC (mgC.L^−1^)	32.5 ± 5.1	27.7 ± 0.8	12.8 ± 2	18.3 ± 3.6
CH_4_ (μM)	202.8 ± 125.3	9.4 ± 4.4	8.4 ± 3.1	412.0 ± 232.1
CO_2_ (μM)	2246.4 ± 730.2	319.1 ± 215.9	348 ± 107.2	2166.7 ± 466.1
N_2_O (nM)	60 ± 20	6 ± 1	80 ± 2	2 ± 2
CO_2_:CH_4_	15.7 ± 11.5	30.4 ± 10.4	55.1 ± 8.2	6.7 ± 3.2

The DOC was highest in soil water, closely followed by the emerging lake (on average 32.5 and 27.7 mg C L^−1^, respectively), while the surface water of the mature lakes presented the lowest DOC concentration (12.8 mg C L^−1^, as compared to 18.3 mg L^−1^ in the hypolimnion) (Table 2). In addition to bulk carbon concentrations, DOM properties of water collected in emerging and mature lakes (although on the following year; Table S1). The emerging lake exhibited approximately twice the amount of chromophoric DOM (a_320_ proxy averaging 165 m^−1^) compared to mature lakes (74 m^−1^ at the surface) and twice the amount of fluorescing DOM (FDOM; F_tot_ proxy averaging 15.5 RU versus 7.0 RU respectively). The relative composition of FDOM fluorophores was similar between both lake types, with a clear dominance of humic-like terrestrial molecules (~81% of all fluorophores). However, DOM was much less chromophoric in the emerging lake (SUVA index 1.2 compared to 2.7 L.mg C^−1^.m^−1^ in mature lakes), and there was more protein-like fluorophores in the emerging lake (particularly amino-acid tyrosine-like).

GHG concentration greatly varied along the transect. Median CO₂ concentrations were highest in soil water and the deep waters of mature lakes, with values of 2246 ± 730 μM and 2167 ± 466 μM, respectively (Table 2). Deep waters in mature lakes showed elevated CH₄ concentrations (412 ± 232 μM) compared to surface waters (8.4 ± 3.1 μM; p = 0.07) and soil water (202.8 ± 125.3 μM, P < .05).

**Figure 2:**
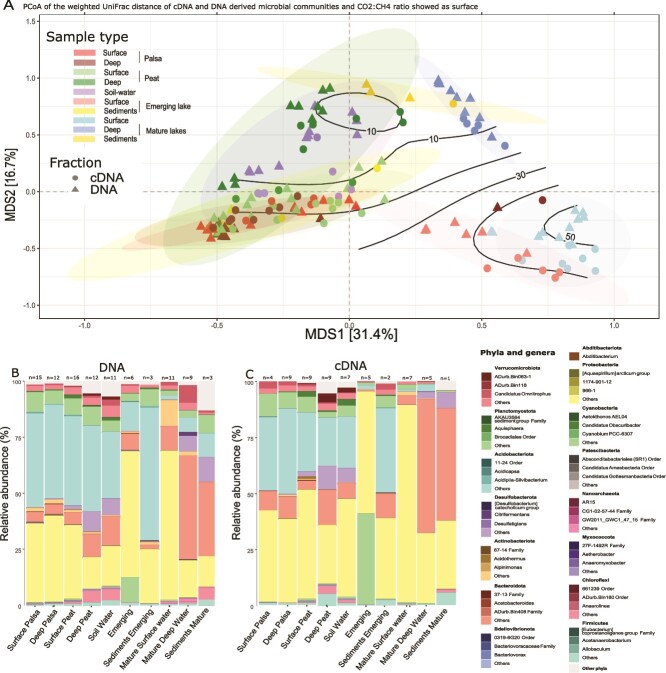
**Bacterial and archaeal beta-diversity and community composition of palsa soils, soil water and lake communities**. In the panel A, the PCoA illustrates the weighted UniFrac distances for the total microbial communities based (DNA-based) and the potentially active communities (cDNA-based) the isolines shows the CO2:CH4 ratio in soil water and lake water samples. Colors refer to sample types. 95% uncertainty ellipses are shown. The relative abundance of the top 15 bacterial and archaeal phyla, of which the three most abundant genera are shown when available, for the total communities based on DNA (B) and the potentially active communities based on cDNA (C).

Emerging lake and surface of mature lake presented similar CH_4_ concentration (9.4 ± 4.4 μM vs 8.4 ± 3.1 μM) (Table 2). But emerging lake presented a lower CO2:CH4 ratio (Fig. 2A, Table 2) compared to mature lake surface (30.4 ± 10.4 vs 55.1 ± 8.2) while soil water and hypolimnion presented much lower ratio (Fig. 2A).

### Microbial community structuring successions from emerging to mature thermokarst lake

**Figure 3:**
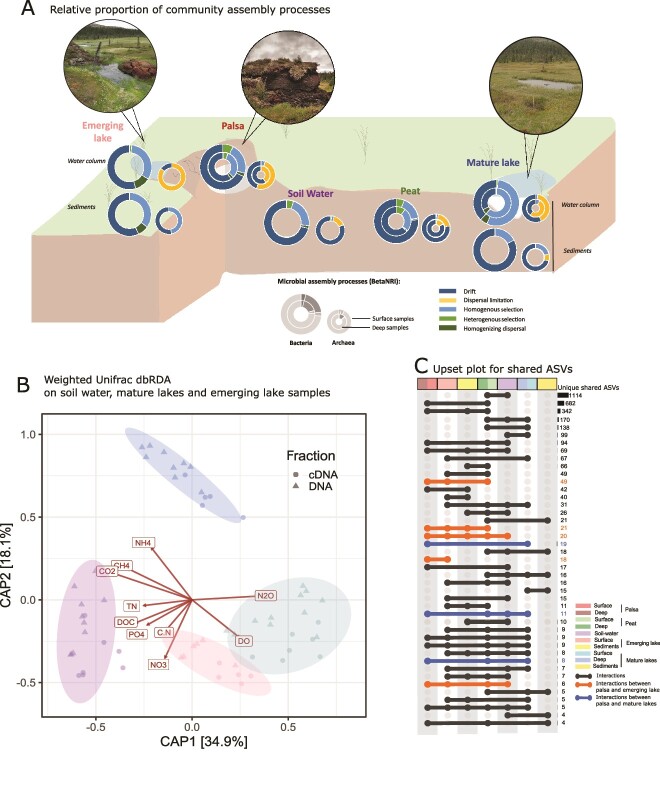
**Microbial connectivity based on ASVs, and community assembly processes from soil to water along a transect of thermokarst formation.** The site landscape photos provide context for the study area. Panel A depicts the proportion of community assembly processes using circle plots: Left circles represent bacteria, and right circles represent archaea. Outer circles correspond to surface samples, while inner circles represent deep samples (hypolimnion or deep peat, or deep palsa). Assembly processes were identified using the Beta Net Relatedness Index (βNRI) and the Raup-Crick (RC) metric within a bin-based approach implemented in iCAMP [49]. Panel B presents dbRDA analysis of water samples, constrained by key environmental variables. The color coding refers to the type of water. Panel C displays an upset plot illustrating shared ASVs among the different units along the transect. Only the top 50 interactions are indicated, and specific interactions highlighted for clarity.

In both palsa and surrounding wetland peat samples, microbial communities at two soil depths showed similar phylogenetic structures (Weighted UniFrac distances), with no significant evolutionary divergence observed (pairwise PERMANOVA, *P* > 0.1) (Fig. 2A). Moreover, while palsa and adjacent wetland peat exhibited close phylogenetic relationships (short Weighted UniFrac distances), their community compositions were similar, though shifts in relative abundances were observed (pairwise PERMANOVA, *P* < .05). Bacterial assemblages were dominated by *Acidobacteriota* (palsa: 42 ± 18%, peat: 39 ± 17%) and *Proteobacteria* (palsa: 36 ± 11%, peat: 24 ± 15%) (Fig. 2C). Palsa archaeal assemblages were predominantly composed by *Crenarcheota* Group 1.1c (85 ± 34% of the archaeal community) (Fig. S1A), notably by *Nitrososphaeria*. Community assembly processes were primarily stochastic, driven by drift (palsa: 67 ± 3%, peat: 70 ± 0.6%), with deeper palsa layers showing more selection processes (Fig. 3A). Archaeal communities were also shaped by stochastic processes, with drift predominant in wetland peat (80 ± 7%) and dispersal limitation in palsa peat (45 ± 19%).

As palsas degrade, emerging lakes form. The microbial communities in the emergent lake appeared at intermediate phylogenetic distances between the surface waters of mature lakes and the deep soils of palsa (Fig. 2A). Community composition differed significantly among emerging lakes, palsa soils, and the surface layer of mature lakes (pairwise PERMANOVA, P < .01), alongside a decreasing CO₂:CH₄ ratio observed from mature lake surface waters to emerging lakes, soil water, and the hypolimnion of mature lakes (Fig. 2A). Furthermore, 114 ASVs were shared between the palsa and the emerging lake, compared to only 38 ASVs shared between the palsa and the mature lake (Fig. 3C).

The emerging lake’s bacterial communities were distinct with that of mature lakes (pairwise PERMANOVA, P < .01), and were dominated by *Proteobacteria* (56 ± 9%)—primarily *Gammaproteobacteria* (32 ± 10%) and *Alphaproteobacteria* (24 ± 3%), alongside *Cyanobacteria* (11 ± 6%) (Fig. 2B). *Acidobacteria,* including *Pseudoacidobacteria,* were also present, and the emerging lake exhibited a higher proportion of *Planctomycetes* (4 ± 0.7%) compared to the mature lakes (Wilcoxon *P* < .05). Archaeal communities were dominated by *Methanoregula* and *Methanobacteria* (Fig. S1A). Flow cytometry further revealed smaller cell sizes (lower FSC and SSC) in the emerging lake (Fig. S3) compared to the mature lakes, further pointing to different communities and phenotypes between them. Bacterial assemblages in the emerging lake were structured mainly by stochastic processes (65 ± 1% for stochastic processes for bacteria and 85 ± 11% for dispersal limitation for archaea) (Fig. 3A). The emerging lake showed the highest proportion of homogenizing dispersal across all transects (bootstrap, p < 0.05), representing 11 ± 5% of the assembly processes. In addition, sediment communities from the emerging lake were closely related phylogenetically to the palsa communities (Fig. 2A) and had a similar composition (Fig. 2C) (pairwise PERMANOVA, P > .05).

In the final stage of permafrost degradation, mature thermokarst lakes that are fully developed and stratified showed distinct community structures between surface and deep waters (pairwise PERMANOVA, P < .01 ) (Fig. 2A). Surface waters were dominated by *Gammaproteobacteria*, *Actinobacteriota*, and *Bacteroidota* (52 ± 8%, 12 ± 4%, and 11 ± 6%, respectively), in particular *Polynucleobacter*, *Limnohabitans*, and members of *Comamonadaceae* and *Methylobacter*. In contrast, deep waters were predominantly composed with *Bacteroidota* (46 ± 13%), specifically *Chlorobia* (37 ± 13%), with additional contributions from *Desulfobacterota* (9 ± 2%) and Candidate phylum *Omnitrophota* (classified as “*Candidatus Omnitrophus”* in *Verrucomicrobia* in Silva database) (9 ± 3%) (Fig. 2B and C). Both the surface and hypolimnetic archaeal communities were dominated by *Nanoarchaeota*, particularly the *Woesearchaeales* order (1 ± 0.6% of the total community) (Fig. S1A). Variations in deep water communities were primarily linked to NH_4_^+^levels (dbRDA, Fig. 3B). In surface and deep waters of mature lakes, bacterial communities were primarily structured by deterministic processes, especially homogenous selection (55 ± 5% and 60 ± 2%, respectively). Sediments in mature lakes were shaped by drift (81 ± 2%) and homogenous selection (19 ± 2%), with archaeal communities showing additional dispersal limitation.

The microbial structure along the chronosequence of thermokarst lake establishment varied greatly. Where in the emerging lake, there was high phylogenetic relatedness to the palsa and dominant stochastic processes, but the mature lakes were highly selective, mainly driven by selective processes. However, only DOC and CH₄ concentrations were significantly correlated with both selective processes, while NO_3_^−^ and CO_2_ concentrations were mainly related to homogeneous selection and heterogenous selection respectively (two-sided mantel test, P < .05).

### Patterns in total and potentially active microbial communities; similarities and differences

The community structure between total (DNA) and potentially active (cDNA) microbial communities had similar general patterns but some differences were observed, as demonstrated by the PCoA results (weighted UniFrac distance, Fig. 2A) and confirmed by PERMANOVA (*P* < .001). Specifically, the potentially active communities exhibited shorter phylogenetic distances, indicating a closer evolutionary relationship, particularly between mature surface lake samples and those from the emerging lake (Fig. 2A).

Notably, the higher relative abundance of the Candidate phylum *Omnitrophota* observed in the DNA fraction of the mature lake hypolimnion was not reflected in the cDNA fraction, where it accounted for less than 1% of the potentially active community (*P* < .01) (Fig. 2D). *Acidobacteria* constituted a significantly larger proportion of the potentially active community in the emerging lake (3 ± 1%) compared to the mature lakes (0.7 ± 0.4%; *P* < .05), despite reaching up to 10% in the DNA fraction of the emerging lake.

### Changes in methanotrophs and methanogens along the transect

Using Buan’s (2018) methanogen classification, methanogenic ASVs from orders such as *Methanobacteriales*, *Methanococcales*, *Methanomicrobiales*, *Methanosarcinales*, *Methanopyrales*, *Methanocellales*, and *Methanomassiliicoccales*, along with families *Methanosarcinaceae* and *Methanosaetaceae*, constituted 0.5 ± 0.2% of the hypolimnion microbial community in mature lakes (0.4 ± 0.3% in cDNA). In emerging lakes, methanogens were less abundant (0.08 ± 0.06%, or 0.09% in cDNA), while soil water samples contained up to 2 ± 1% methanogens (1.4 ± 0.8% in cDNA). In sediments, methanogens represented 0.3 ± 0.4% (0.1% in cDNA) in emerging lakes and 2 ± 1.8% in mature lakes. Methanogenic communities in oxic environments were dominated by *Methanoregula* and *Methanobacterium.* In contrast, wetland peat harbored a more diverse methanogen assemblage, with abundant Rice cluster II family ASVs (52 ± 41%), which were absent from the lake samples (Fig. S1A). The hypolimnion contained the highest proportion of *Methanosaeta* (15 ± 13%). Finally, *Methanomassiliicoccaceae* ASVs were observed across wetland peat, soil water, the hypolimnion of mature lakes, and sediments of emerging lake (Fig. S1A).

Following Guerrero-Cruz et al. (2021), methanotrophic taxa—ASVs from families including *Methylococcaceae*, *Methylothermaceae*, *Methylocystaceae*, *Beijerinckiaceae*, *Methylacidiphilaceae*, *Methanosarciniales*, *Methylacidimicrobium*, and *Methylomirabilaceae*—accounted for 5 ± 3% (4 ± 1% in cDNA) of the microbial community in emerging lake surface waters, and 4 ± 3% (3 ± 3% in cDNA) in mature lake surface waters. Methanotrophs were less abundant in the mature lake hypolimnion (1 ± 1%, or 2 ± 1% in cDNA) but included anaerobic methanotrophs such as *Methylomirabilota*. Methanothrophs reached 4 ± 2% (12 ± 7% in cDNA) in wetland peat. Methanotrophic community composition shifted from a dominance of *Proteobacteria*, including some *Verrucomicrobia* ASVs, to a more diverse assemblage in wetland peat, soil water, and the hypolimnion of mature lakes (Fig. S1B). In these locations, the *Methylomirabilota* ASV Sh765B-TzT-35 constituted a significant portion of methanotrophs, ranging from 12 ± 9% (1 ± 0.8% of the total microbial population) in soil water to 80% in sediments, although total methanotrophic abundance in sediments remained low. Additionally, *Candidatus Methanoperedens* was detected in the sediments of mature lakes.

## Discussion

### Shifts in community assemblages along the chronosequence of thermokarst lake formation

Microbial communities were studied using a space-for-time approach to investigate the impact of palsa degradation and thermokarst lake development on microbial structure and their role in GHG dynamics. As expected, the microbial composition of the sediments of the emerging lake was very similar to the microbial composition of the palsa reflecting the collapse of palsa soils as its permafrost core thaws [50]. Moreover, the emerging lake water was enriched in *Acidobacteriota,* a phylum typical of acidic soil [51, 52] and previously found in this peatland region [53]. We found this phylum in the peat, palsa, and soil water, and it is present and active in the emerging lake but rather absent from the mature lake. This suggests that some factor is overriding the selection processes in the emerging lake or that they lack fitness to survive in the mature lakes. These findings, along with the higher number of shared ASVs (3 times more ASV shared between the palsa and the emerging lake than between the palsa and the mature lake), underscore the strong microbial connectivity between the palsa and the emerging lake, likely driven by a mass effect from palsa-associated microbes. The shared ASVs and short UniFrac distances also suggest common evolutionary histories. Immigration is known to increase stochasticity, particularly through homogenizing dispersal [54]. Here the microbial connectivity could loosen the selection that we observed in the other lakes, as it was previously described in Lindström and Langenheder, (2012) and Evans et al., (2017) [22, 55]. Consequently and in addition to results of carbon connectivity and differentiated carbon degradation processes from Peura et al. (2020) [14], we suggest that in the early stages of thermokarst development, eroded soils and palsa subsidence are an important vector of microbes.

Additionally, the dbRDA revealed a strong influence of nitrogen species on microbial communities (highest loading on the CAP2 axis; Fig. 3B). The elevated NO_3_^−^ concentration in the emerging lake likely contributed to homogeneous selection, as indicated by the two-sided Mantel test, where NO_3_^−^ was significantly correlated with this assembly process (P < .05). *Pseudoacidobacteria* are known for their ability to reduce nitrate and nitrite from their genes encoding nitrate reductase [56, 57]. Similarly, *Acidobacteria* can utilize nitrite, in addition to metabolizing various organic and inorganic nitrogen species [51, 58]. The emerging lake was also associated with a higher proportion of an annamox bacteria, *Planctomycetes* [59, 60], a taxon also present in the palsa and soil water, further highlighting the connectivity between these environments. The lower FSC and SSC values observed in flow cytometry (Fig. S2) for microbial populations from the emerging lake suggest smaller cell sizes and reduced intracellular complexity [61]. This adaptation would lead to a higher surface-to-volume ratio of the cells that enhances nutrient uptake efficiency [62], particularly given the lower NH_4_^+^ concentrations in the emerging lake (30 μg N.L^−1^ compared to 66 μg N.L^−1^ at the surface of the mature lake). The different communities and morphologies observed here suggest different phenotypes and nutrient uptake strategies between the emerging and mature lakes.

Overall, deterministic processes have been shown to dominate the assembly of subarctic thermokarst lake communities [20]. Here, we build on this finding by demonstrating that during thermokarst lake formation, community assembly initially involves predominantly stochastic processes, driven by the strong connectivity with surrounding soils. However, selective pressures, particularly for nitrogen cycle players, arise early on. Over time, deterministic assembly processes become more prominent as environmental filtering takes over.

### Implications for GHG emissions of microbial successions during thermokarst lake formation

Methanogenic and methanotrophic communities have been extensively studied in mature thermokarst systems [12, 19, 53, 63, 64]. However, there is limited information on their succession and assembly processes over time. Here, ASVs associated with known methanogens were detected across all transects, with notable shifts in taxonomic composition corresponding to habitat transitions highlighted with high dispersal limitation for Archaea. *Methanoregula*, an acidophilic methanogen adapted to low nutrient conditions [65], and *Methanobacterium* both dominated in the palsa, the emerging lake and its sediments, as well as the surface water of mature lakes, facilitated by surface connectivity. In contrast, the methanogenic community became more diverse, notably with acetoclastic methanogens such as *Methanosaeta [**66**]*, and methylotrophic methanogens like *Methanomassiliicoccaceae [**67**]* increasing in abundance in peat and the hypolimnion. This shift likely reflects changes in DOM quality [14], as supported by DOM data collected on the following year (SI). Notably, results indicate larger amounts of DOM (DOC, CDOM, FDOM), smaller molecules (S_285_), less colored DOM (SUVA index), more protein-like molecules and more aromaticity in the emerging lake (Table 1, Table S2 and Peura et al. (2020))**.** Hence the diversity of methanogens and their shift along the transects can come from diverse sources of DOM of different ages and nature. This diversity enables continuous CH_4_ production through a portfolio effect, with varied consequences for climate feedback loops depending on DOM age. Resolving the specific pathways linking DOM degradation to GHG dynamics will require metagenomic analysis, which will be addressed in a companion study.

In soil water, methanogens constituted ~50% of the archaeal community (Fig. S1A), with a dominance of Rice Cluster II, as previously found in thawing permafrost soils [68]. In the present study, soil water and the hypolimnion of mature lakes presented the highest concentrations of CO_2_ and CH_4_, and the lowest CO_2_:CH_4_ ratios (Table 1). These habitats also had the highest relative abundance of methanogen ASVs. These results are reflecting the high potential for methanogenesis in both locations, where anoxic conditions prevails and exchange with the atmosphere is restricted. Moreover, the highest CH_4_ concentrations were observed in areas with the most diverse methanogen communities (Fig. S1A), aligning with findings from previous studies in boreal peatland environments [64].

*Acidobacteria,* particularly abundant in the emerging lake, are known producers of acetate [69]. However, we found no known acetoclastic methanogens in the water and sediments of the emerging lake, where the archaeal community was largely dominated by hydrogenotrophic methanogens, as previously observed in this valley by Crevecoeur et al. (2016). These observations suggest that the environmental conditions during thermokarst lake formation favored hydrogenotrophic methanogens, even in the presence of acetate producers. It may indicate that viable hydrogenotrophic methanogens may be present in oxygenated conditions as previously observed in arctic thermokarst lake and capable of quickly adjusting their metabolism as these shallow water bodies undergo diurnal phases of stratification and mixing depending on meteorological conditions [12]. Or that methanogens are submit to more dispersal limitation, leading to differentiated methanogenesis along the transect. On-going companion metagenomic studies in this system will provide valuable insights in this proposed scenario.

We also identified a clear link between assembly processes and the GHG concentrations, with CH_4_ significantly contributing to both homogeneous and heterogeneous selection, while CO_2_ was primarily associated with heterogeneous selection. This likely accounts for the observed differences in CH_4_ cycling community composition described above. Indeed, the CO_2_:CH_4_ ratio corresponds with the observed community dissimilarities, as CH_4_ and CO_2_ dynamics are influenced by both methanogenesis, acetotrophy, organic matter degradation and methanotrophy, which vary along the transect. However, it is important to note that other biological and physical mechanisms also influence GHG concentrations and CO_2_:CH_4_ ratios, including organic matter biodegradation pathways, photodegradation, and water column mixing (i.e. GHG venting to the atmosphere). Although DOC concentration was greater in the emerging lake, which might typically lead to increased CO_2_ production through bacterial respiration, the CO_2_:CH_4_ ratio was lower than that of the mature lake surface. This discrepancy may be attributed to either enhanced CH_4_ production and CO_2_ consumption by *Methanoregula* (a hydrogenotrophic methanogen) during periods of oxygen limitation in the emerging lake, or reduced CH_4_ conversion to CO_2_ by the methanotrophic community in the presence of oxygen and photo inhibiting conditions that might occurs in this type of lakes [70].

Data on total community composition (based on DNA templates) indicate high proportions of symbiotic Bacteria and Archaea along our study transects, particularly in the anoxic hypolimnion of mature lakes. Notably, this includes members of the candidate phylum *Omnitrophota*, which is characterized as a symbiotic acetate-producing nanobacterium [71]. The hypolimnion was also enriched with acetoclastic methanogens such as *Methanosaeta,* suggesting the presence of a consortium or syntrophy involved in CH_4_ production via this pathway. However, this was not clearly confirmed with the cDNA template, as the relative abundance of candidate phylum *Omnitrophota* was reduced in the hypolimnion, although some potential activity was still observed. The *Woesearchaeales* order*,* an anaerobic archaeal taxon with a syntrophic lifestyle that may interact with methanogens through syntrophic relationships [72], was the dominant archaeal group in mature lakes. Further reinforcing the idea of symbiosis for CH_4_ production that can help sustain a diverse methanogenic community.

### Conclusions and future directions

Distinct patterns of microbial community assembly were evident along the chronosequence of thermokarst lake formation in a subarctic peatland. The degradation of permafrost mounds leads to the formation of emerging lakes characterized by distinct microbial communities compared to mature lakes. These communities were shaped by mass effects for certain taxa and nitrogen availability for others. As emerging lakes transition into well-structured thermokarst lakes, selective processes dominate, fostering diverse and active communities of methanogens, methanotrophs, and organic matter degraders. The selective assembly processes are driven by environmental factors, particularly CH_4_, DOC, and NO_3_^−^ concentrations. Further research is necessary to elucidate the complex interactions between nitrogen and carbon metabolisms in these nitrogen-limited ecosystems, as continued warming accelerates permafrost degradation.

## Supplementary Material

Supplementary_information_Leroy_et_al_ycaf027

## Data Availability

Unprocessed sequences for DNA and cDNA templates are available at Sequence Read Archive and are accessible under the bioproject ID PRJNA1101881.
