# Supplementary material for: Terrestrial-aquatic connectivity structures microbial communities during the formation of thermokarst lakes
Source: ISME Commun. 2025 Feb 10;5(1):ycaf027. doi: 10.1093/ismeco/ycaf027 (PMC11879182; doi:10.1093/ismeco/ycaf027)
Supplement: Supplementary_information_Leroy_et_al_ycaf027 [file supplementary_information_leroy_et_al_ycaf027.pdf]

**Supplementary information Leroy *et al.* : "Terrestrial-aquatic connectivity structures microbial communities during the formation of thermokarst lakes'**

***Changes in microbial populations along thermokarst formation assessed by flow cytometry***

Microbial populations from lake and soil-water samples were characterized using a BD C6 Accuri Cytometer (BD, USA). Samples were stored in polypropylene tubes and cells were fixed with 1% glutaraldehyde (final concentration) and kept frozen at -80°C until analysis. Unfrozen samples were then stained with SyberGreen (2.5x final concentration [1]) for 15 minutes. Stained 0.2-µm filtered samples served as blanks. Flow cytometry data analysis was conducted in R using the FlowCore [2] and CytoExploreR [3] packages. Gating was performed on 25,000 events, with forward scatter (FSC) used as a proxy for cell size and side scatter (SSC) as an indicator of intracellular complexity.

***Greenhouse gas sampling and calculation of dissolved concentrations***

Dissolved gas concentrations of carbon dioxide ($CO_2$), methane ($CH_4$) and nitrous oxide ($N_2O$) were measured in the hypolimnion and epilimnion of thermokarst lakes, and in surface soil water along the transects using the headspace equilibration technique [4]. Briefly, plastic syringes were used to collect 120 mL of epilimnetic lake water (at ~0.25 m depth) or soil water, equilibrated with 20 mL of atmospheric air, and then shaken vigorously for 5 minutes. For gas equilibration of hypolimnetic lake samples, the water was pumped from ~2 m-depth into a 2-L HDPE bottle modified with a port to attach a syringe at the bottom. A headspace of 32 mL of atmosphere was introduced into the bottle using the attached syringe (filling with water), also shaken for 5 minutes to equilibrate. The equilibrated headspace was injected into 12 mL Exetainer vials (Labco, UK) preflushed with $N_2$ and vacuumed. Water temperature was taken at the beginning and end of the equilibrium to account for gas solubility using Henry's law. Analysis was done by gas chromatography: quantification was done using a Thermo 1310 gas chromatograph equipped with a TRI-Plus Head-Space auto-sampler (Thermo Fisher Scientific Inc., Massachusetts, USA). Five mL of gas were injected and separated into two injection loops. One loop was used to quantify CO2 and CH4 (separated with two columns in series, HSQ 80/100 10' x 1/16'' and MS 5A 6' x 1/16'') with a thermal conductivity detector (TCD) and a flame ionization detector (FID). Quantification of N2O on the second

loop (separated with a HSQ 80/100 4' x 1/16'' column) was obtained with an electron capture detector (ECD; P5 for makeup gas). The resulting aqueous dissolved gas concentrations were calculated using air pressure, temperature at equilibration, temperature of water at collection, and temperature-dependent Henry's law constants [5]. By subtracting the gas concentration in the water at equilibrium with the atmosphere. Atmospheric samples were also collected in Exetainers to use as a baseline of atmospheric GHG concentration and calculate dissolved gas concentrations more accurately. Calculations were completed in R (Version 4.2.3) using the open-source code from the National Science Foundation's National Ecological Observatory Network published under GNU Affero General Public License [6].

***Microbial populations observation by flow cytometry***

Two populations could be identified in the cytograms (**Error! Reference source not found.**). The first one was characterized by a side scatter value higher than 4 (on a $Log10$ scale, population 1), and the second one was defined by its lower side scatter and forward scatter values (population 2). Shifts in the abundance and shape of these populations were observed between the emerging lake and the mature lakes (**Error! Reference source not found.**). Specifically, the emerging pond presented lower forward scatter values (median of 3.7 $log10$) by comparison to median values of 4.5 and 4.8 for surface and deep water of the mature ponds.

***Dissolved organic matter composition***

DOC concentrations were measured in August 2021, concurrently with microbial DNA sampling. DOM samples were collected in August 2022 from the same locations in the emerging and surface mature lakes as those sampled for DOC and microbial DNA the previous year. This was necessitated by analytical issues with the DOM samples taken in 2021. Although quantitative variations between years are expected, we assume that qualitative characteristics of DOM remained consistent among the years. Lake samples were filtered onto glass fiber filters (GF75, AMD Manufacturing Inc., Ontario, Canada) and kept in amber glass vials in the dark at 4°C, until analyses within 20 days of collection.

Absorbance spectra were run from 200 to 800 nm using 1-cm quartz cuvettes on a dual-beam spectrophotometer (UV-Vis, Agilent, California, USA), from which was

calculated the absorption coefficient at 320 nm ($a_{320}$), a proxy of chromophoric DOM concentration (CDOM). The SUVA index was calculated with the CDOM R package [7] as the specific absorbance at 254 nm divided by DOC concentration, and used as a proxy for DOM aromatic content. The  absorption slope between 275 and 295 nm ($nm^{-1}$), $S_{285}$, was used as proxy of the molecular size of DOM [8].

To characterize the composition of fluorescent DOM (FDOM) in water samples, excitation-emission matrices of fluorescence (EEMs) were obtained on a spectrofluorometer (Cary Eclipse, Agilent, California, USA) for an excitation range from 240 to 450 nm, emission range from 300 to 560 nm, and a slit width of 2 nm. Blank-subtracted EEMs were corrected for inner-filter effect and standardized to Raman units (RU) after the excision of Raman and Rayleigh scatter peaks. The principal fluorescent components were extracted using an 8-components model developed with Parallel Factor Analysis (PARAFAC) [9] on thermokarst lakes and ponds of the Canadian Arctic By Pacoureau et al (2023) [10] The model was developed on 365 EEMs from using data from 22 thermokarst lakes and ponds in the Arctic, as well as leachates from benthic mats, surrounding terrestrial material, and fresh vegetation. PARAFAC modelling was performed according to Stedmon and Bro (2008) using the Matlab drEEM toolbox [11]. Component comparison to databases and quantification of fluorophores were made as described in SI of Preskenis [12]. The model comprises four humic-like terrestrial components (HT1-4), two humic-like microbial components (HM1-2) and 2 protein-like components (P1-2). The sum of the maximum fluorescence by each eight components ($F_{tot}$) was used as a proxy for FDOM quantity.

**Table S1**: DOM composition in lake water, including the absorbance at 254 nm normalized per unit DOC (SUVA), the absorption slope calculated from 275 to 295 nm ($S_{285}$), the humic-like terrestrial fluorophores (HT), the humic-like microbial fluorophores (HM), and the protein-like fluorophores (P), all given in relative percentage of total fluorescence ($F_{tot}$) and in Raman units (RU).

| | Emerging lake | | Mature lakes epilimnion | |
|---|---|---|---|---|
| SUVA ($L.mgC^{-1}m^{-1}$) | 1.2±0.03 | | 2.7±1.3 | |
| $S_{285}$ ($nm^{-1}$) | 0.012 | | 0.006 ± 0.001 | |
| $a_{320}$ ($m^{-1}$) | 165 | | 74±17 | |
| | % | RU | % | RU |
| $F_{tot}$ | - | 15.5 | - | 7.0±2.2 |
| HT1 | 29.4 | 4.6 | 28.6±0.7 | 2.0±0.7 |
| HT2 | 12.9 | 2 | 15.2±0.1 | 1.0±0.2 |
| HT3 | 37.3 | 5.8 | 35.7±0.3 | 2.5±0.8 |
| HT4 | 0.94 | 0.1 | 1.1±0.7 | 0.5±0.1 |
| HM1 | 1.1 | 0.2 | 3±0.3 | 0.2±0.1 |
| HM2 | 3.5 | 0.5 | 3.8±0.2 | 0.3±0.1 |
| P1 | 4.8 | 0.8 | 5.5±0.3 | 0.4±0.1 |
| P2 | 9.8 | 1.5 | 6.8±0.5 | 0.5±0.2 |

***Supplémentary information on statistical approaches***

To address concerns regarding overinterpretation and to increase statistical power, two PERMANOVAs were performed directly on the Weighted Unifrac distance matrices, following the approach recommended by Buttigieg and Ramette (2014) [13]. The first PERMANOVA included all samples, with transect location and depth as explanatory variables. The second analysis focused on lake and soil-water samples, testing all possible combinations of environmental variables. The AIC criterion was used to discriminate between models, with AIC values calculated using the *AICcPermanova* package [14]. The model with the lowest AICc was selected as the best explanatory model, and the model retained included DO, $NH_4$, $NO_3$, $PO_4$, TN, $CH_4$, $CO_2$, DOC, C/N. Permutation test for adonis under reduced model for water samples was performed with a number of permutations of 9999.

**Table S2:** result of the PERMANOVA on water sample

| | Df | SumOfSqs | R2 | F | Pr(>F) |
|---|---|---|---|---|---|
| DO | 1 | 0.24971 | 0.15190 | 25.2050 | 0.0001 *** |
| NH4_ug_N.L | 1 | 0.10565 | 0.06427 | 10.6639 | 0.0001 *** |
| NO3_ug_N.L | 1 | 0.35794 | 0.21773 | 36.1292 | 0.0001 *** |
| PO4_ug_N.L | 1 | 0.09450 | 0.05748 | 9.5381 | 0.0001 *** |
| TN_ug_N.L | 1 | 0.12922 | 0.07860 | 13.0430 | 0.0001 *** |
| CH4 | 1 | 0.02888 | 0.01757 | 2.9151 | 0.0183 * |
| CO2 | 1 | 0.03825 | 0.02327 | 3.8613 | 0.0017 ** |
| DOC | 1 | 0.08370 | 0.05091 | 8.4480 | 0.0001 *** |
| C.N | 1 | 0.05085 | 0.03093 | 5.1322 | 0.0010 *** |
| Residual | 51 | 0.50527 | 0.30735 | | |
| Total | 60 | 1.64395 | 1.00000 | | |

Signif. codes: 0 ‘***’ 0.001 ‘**’ 0.01 ‘*’ 0.05 ‘.’ 0.1 ‘ ’ 1

**dbRDA on water samples**

The dbRDA was performed on water and soil water samples with *capscale* function in *vegan* package (constraint variables were : DO, $NH_4$, $NO_3$, $PO_4$, TN, $CH_4$, $CO_2$, DOC, N2O, C/N

**Table S3 :** Eigvalues for the 2 retained axis

| | CAP1 | CAP2 |
|---|---|---|
| Eigvalues | 1.132551709 | 0.504423096 |

**Table S4:** Partitioning of squared weighted Unifrac distance

| | Inertia | Proportion | Rank |
|---|---|---|---|
| Total | 2.76912 | | |
| RealTotal | 2.80038 | 1.00000 | |
| Constrained | 2.13968 | 0.76407 | 10 |
| Unconstrained | 0.66070 | 0.23593 | 26 |

**Table S4:** Coefficient associated with explanatory variables for the dbRDA axis CAP1 and CAP2 (biplot)

| | CAP1 | CAP2 |
|---|---|---|
| DO | 0.26594 | -0.5762 |
| NH4_ug_N.L | -0.4168 | 0.7159 |
| NO3_ug_N.L | -0.3503 | -0.7932 |
| PO4_ug_N.L | -0.463 | -0.2373 |
| TN_ug_N.L | -0.5193 | -0.033 |
| CH4 | -0.7235 | 0.36601 |
| CO2 | -0.876 | 0.39336 |
| N2O | 0.93416 | -0.0314 |
| DOC | -0.7285 | -0.4376 |
| C.N | -0.19299 | -0.27445 |

**Supplementary figures Leroy *et al.* : "Terrestrial-aquatic connectivity structures microbial communities during the formation of thermokarst lakes**

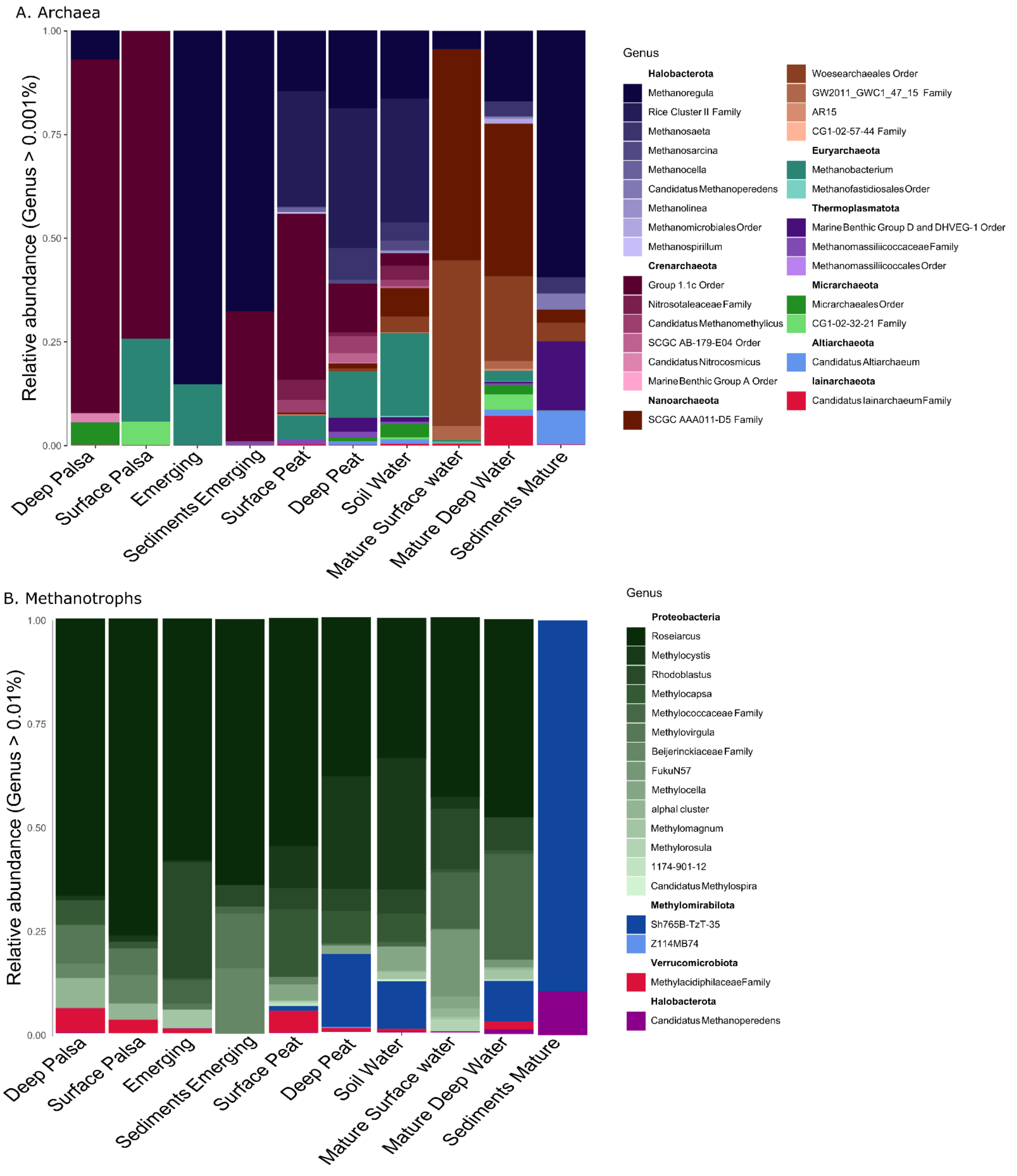


**Fig. S1:** Relative abundance of ASVs in Archaea (A) and known methanotrophic taxa (B).

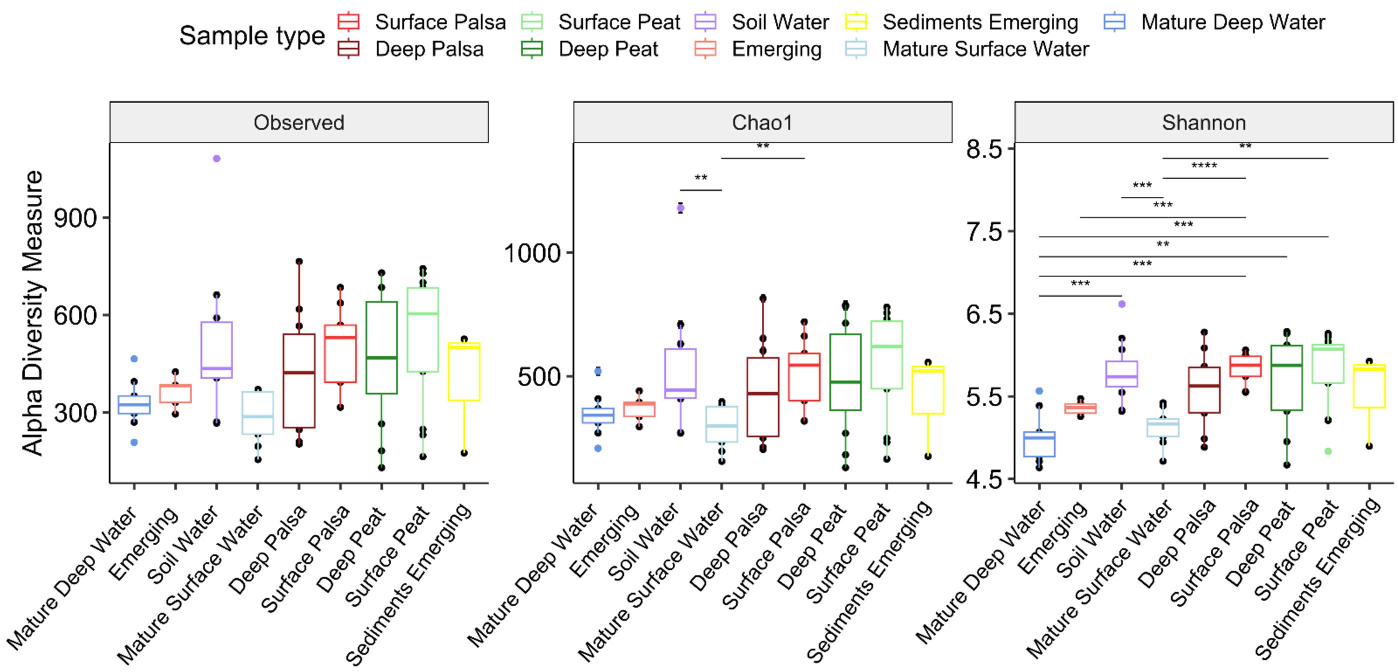


**Fig. S2:** Alpha diversity indices (Observed, Chao1, Shannon) across each part of the transect. Significant differences, calculated by Wilcoxon test with Benjamini-Hochberg correction, are indicated as follows: $p < 0.05$ (*), *p < 0.01 (**), p < 0.001 (***)*.

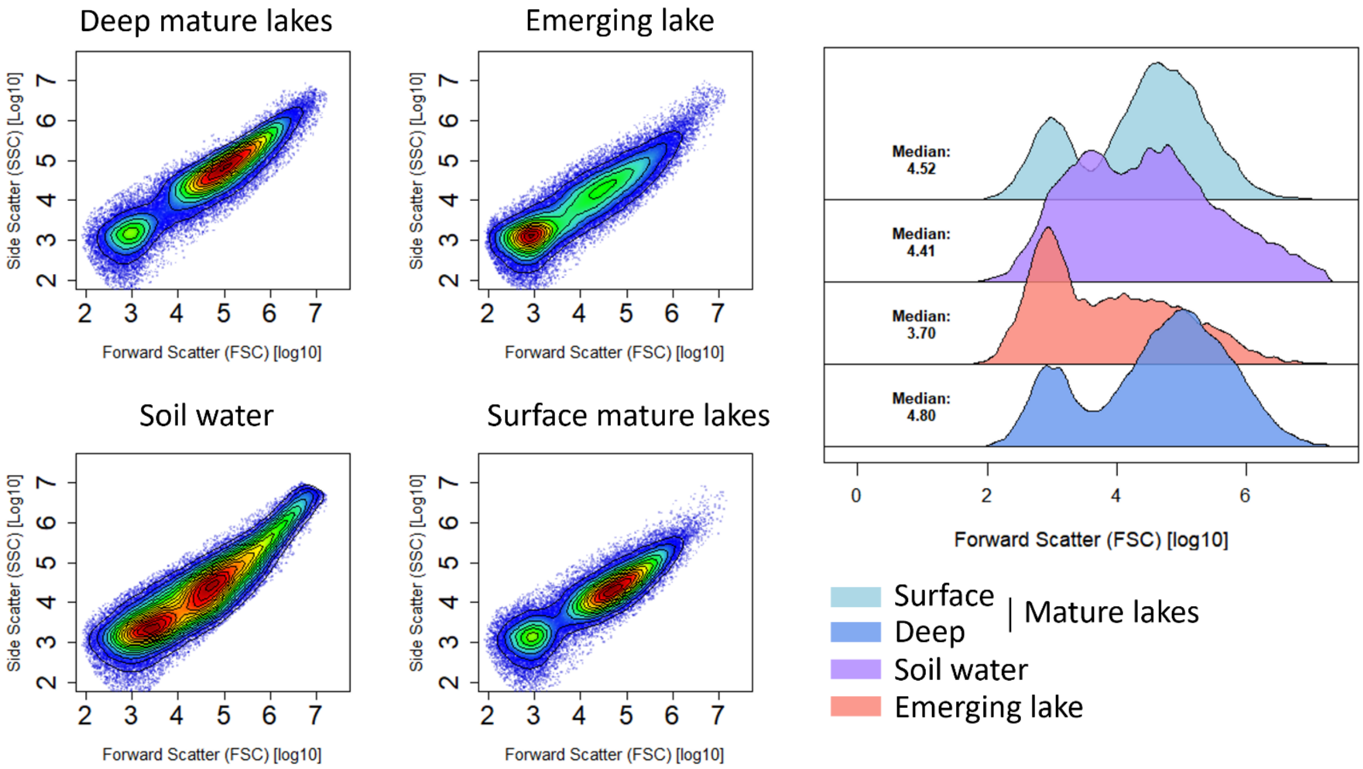


**Fig. S3:** Microbial flow cytometry profiles (left) and fluorescence intensity of forward scatter (FSC) (right) for microbial populations in water samples (n = 3 for both the emerging lake and soil water, n = 6 for both the surface and the hypolimnion of mature lakes). Populations are displayed with forward scatter (FSC) plotted against side scatter (SSC).

## REFERENCES


1. Vignola M, Werner D, Hammes F, King LC, Davenport RJ. Flow-cytometric quantification of microbial cells on sand from water biofilters. *Water Research* 2018; **143**: 66–76.
2. Hahne F, LeMeur N, Brinkman RR, Ellis B, Haaland P, Sarkar D, et al. flowCore: a Bioconductor package for high throughput flow cytometry. *BMC Bioinformatics* 2009; **10**: 106.
3. Hammill D. CytoExploreR. 2023.
4. Kampbell DH, Wilson JT, Vandegrift SA. Dissolved Oxygen and Methane in Water by a GC Headspace Equilibration Technique. *International Journal of Environmental Analytical Chemistry* 1989; **36**: 249–257.
5. Sander R. Compilation of Henry's law constants (version 4.0) for water as solvent. *Atmospheric Chemistry and Physics* 2015; **15**: 4399–4981.
6. Aho K, Cawley K, DelVecchia A, Stanley E, Raymond P. Dissolved greenhouse gas concentrations derived from the NEON dissolved gases in surface water data product (DP1.20097.001). 2021. [object Object].
7. Massicotte P, Markager S. Using a Gaussian decomposition approach to model absorption spectra of chromophoric dissolved organic matter. *Marine Chemistry* 2016; **180**: 24–32.

8. Laurion I, Massicotte P, Mazoyer F, Negandhi K, Mladenov N. Weak mineralization despite strong processing of dissolved organic matter in Eastern Arctic tundra ponds. *Limnology and Oceanography* 2021; **66**: S47–S63.
9. Bro R. PARAFAC. Tutorial and applications. *Chemometrics and Intelligent Laboratory Systems* 1997; **38**: 149–171.
10. Pacoureau T. Caractérisation de la matière organique dissoute et de sa dégradation bactérienne dans les mares de la toundra polygonale à coins de glace. 2023. phd, Université du Québec, Institut national de la recherche scientifique.
11. R. Murphy K, A. Stedmon C, Graeber D, Bro R. Fluorescence spectroscopy and multi-way techniques. PARAFAC. *Analytical Methods* 2013; **5**: 6557–6566.
12. Prėskienis V, Laurion I, Bouchard F, Douglas PM, Billett MF, Fortier D, et al. Seasonal Patterns in Greenhouse Gas Emissions from Lakes and Ponds in a High Arctic Polygonal Landscape. *Limnology and Oceanography* 2021; **66**: S117–S141.
13. Buttigieg PL, Ramette A. A guide to statistical analysis in microbial ecology: a community-focused, living review of multivariate data analyses. *FEMS Microbiology Ecology* 2014; **90**: 543–550.
14. Corcoran D. AICcPermanova: Model Selection of PERMANOVA Models Using AICc. 2023. , 0.0.2
